# Combined 23-Gauge Transconjunctival Sutureless Vitrectomy and Cataract Surgery in Cases with Cataract and Posterior Segment Diseases

**DOI:** 10.4103/0974-9233.71602

**Published:** 2010

**Authors:** Ahmet Taylan Yazici, Necip Kara, Ercument Bozkurt, Mehmet Cakir, Hasan Goker, Ahmet Demirok, Omer Faruk Yilmaz

**Affiliations:** Istanbul Beyoglu Eye Research and Training Hospital, Galata, Istanbul, Turkey

**Keywords:** 23-Gauge Transconjunctival Sutureless Vitrectomy, Cataract Surgery, Postoperative Hypotony

## Abstract

**Background:**

Combined cataract surgery and transconjunctival sutureless vitrectomy are a good option in patients with cataract and vitreoretinal diseases.

**Aim:**

To evaluate the effectiveness, outcomes, and complications of combined 23-gauge transconjunctival sutureless vitrectomy and cataract surgery.

**Settings and Design:**

A retrospective case series was conducted at the Beyoglu Eye Education and Research Hospital.

**Materials and Methods:**

In this study, 28 eyes of 28 patients underwent combined 23-gauge transconjunctival sutureless vitrectomy and phacoemulsification and IOL implantation for cataract and various posterior segment diseases. The outcome measures included, visual acuity, intraocular pressure changes, and anatomical success were evaluated.

**Results:**

The mean follow-up was 4.8 months (range, 3-15 months). Mean overall preoperative visual acuity was 20/333, and final acuity was 20/95 (*P* < 0.001). Mean intraocular pressure (IOP) on the preoperative and first postoperative day was 15.6 ± 7.5 and 13.8 ± 3.3 mmHg, respectively (*P* > 0.05). Three eyes (10.7%) had postoperative hypotony (<6 mmHg)that all recovered spontaneously within the first postoperative week. Three eyes (10.7%) required laser treatment for iatrogenic retinal tears. Anatomical success was obtained in all cases. No serious complications such as endophthalmitis were observed during the follow-up period.

**Conclusion:**

Combined transconjunctival sutureless vitrectomy and phacoemulsification was effective and safe in patients with significant lens opacities and vitreoretinal pathology. Although the anatomic and visual outcomes were satisfactory, the outcomes depended mainly on underlying vitreoretinal pathology.

## INTRODUCTION

Fujii *et al*. introduced 25-Gauge (G) transconjunctival sutureless vitrectomy (TSV) in 2002 following a self-sealing pars plana sclerotomy that was described by Chen in 1996.^1^,^2^ Usage of 25-G TSV presented some problems including the significant flexibility of the instruments resulting in reduced manipulation, inadequate illumination, and reduced lumen of the microcannulae for fluid flow.^3^–^5^ In 2005, Eckardt described a 23-G transconjunctival system to overcome limitations of 25-G TSV.^6^

Transconjunctival sutureless vitrectomy provides many advantages including a sutureless smaller surgical incision, faster wound healing, reduced surgical time, less postoperative inflammation, reduced induction of postoperative astigmatism, better patient comfort, and faster patient convalescence.^3^,^7^

Cataract formation and vitreoretinal diseases can occur together, especially in the elderly population. Intraoperative visualization of the posterior segment during vitrectomy can be affected by lens opacity. In addition, if the cataract is not significant at the time of vitrectomy, vitreoretinal surgery, and usage of an intraocular tamponade can accelerate the process of cataract formation.^8^

With the development of clear corneal phacoemulsification and intraocular lens implantation, combined vitreoretinal and cataract surgery is commonly used as an effective and safe procedure in selected cases.^9^

We report the outcomes of the combined 23-G TSV and cataract surgery in cases with various vitreoretinal diseases.

## MATERIALS AND METHODS

We retrospectively reviewed the medical records of the 28 eyes of 28 patients who underwent 23-G TSV combined with phacoemulsification and posterior chamber intraocular lens implantation. All surgical cases were performed by the same surgeons (ATY, MC) in Beyoglu Eye Education and Research Hospital, in Istanbul, Turkey between January 2008 and February 2009. The study protocol was reviewed and approved by the Institutional Ethics Committee of Beyoglu Eye Education and Research Hospital. Informed consent was obtained from all of the subjects before the surgery. Mean follow-up was 4.8 months (range, 3–15 months).

Demographic data including age, gender, operative eye, and indication for vitreoretinal surgery were collected [Table 1]. All subjects had complete preoperative and postoperative examinations including measurement of Snellen best corrected visual acuity (BCVA), slitlamp biomicroscopy, intraocular pressure (IOP) measurement using applanation tonometry, posterior segment visualization by indirect ophthalmoscopy, and ultrasonography when the fundus could not be visualized.

**Table 1:** Details of cases that underwent combined phacoemulsification and vitreoretinal surgery

Case no	Gender	Age	Diagnosis	Operative procedures	Endotamponade	Preoperative BCVA	Postoperative BCVA
1	F	71	VH	PHACO + PCIOL +23-G-TSV+RT	SO-1000 cSt	HM	20/200
2	M	70	ERM	PHACO + PCIOL + 23-G-TSV + ERMP + İLMP	SF_6_	20/50	20/50
3	M	60	RD	PHACO + PCIOL + 23-G-TSV + EL	C_3_F_8_	HM	LP
4	F	74	VH	PHACO + PCIOL + 23-G-TSV	No	HM	20/30
5	F	69	RS	PHACO + PCIOL + 23-G-TSV +EL	C_3_F_8_	20/100	20/60
6	M	64	TRD	PHACO + PCIOL + 23-G-TSV +MP	SO-5000 cSt	HM	CF
7	M	65	ERM	PHACO + PCIOL + 23-G-TSV + ERMP + İLMP + EL	Air	20/200	20/100
8	M	59	TRD	PHACO + PCIOL + 23-G-TSV + MP + EL	SO-1000 cSt	20/200	HM
9	F	57	ERM	PHACO + PCIOL + 23-G-TSV + ERMP	Air	20/200	20/200
10	F	71	ERM	PHACO + PCIOL + 23-G-TSV + ERMP	Air	20/200	20/50
11	F	51	TRD	PHACO + PCIOL + 23-G-TSV + MP + EL	SO-5000 cSt	CF	HM
12	F	62	ERM	PHACO + PCIOL + 23-G-TSV + ERMP + İLMP	No	20/100	20/60
13	M	74	ERM	PHACO + PCIOL + 23-G-TSV + ERMP + İLMP	No	20/200	20/30
14	F	47	TRD	PHACO + PCIOL + 23-G-TSV + MP + EL	SF_6_	HM	20/200
15	M	57	RD	PHACO + PCIOL + 23-G-TSV + EL	SO-1000 cSt	HM	20/200
16	M	28	IOFB	PHACO + PCIOL + 23-G-TSV + IOFBR + EL	No	20/40	20/25
17	F	66	VH	PHACO + PCIOL + 23-G-TSV + MP + EL	Air	LP	CF
18	F	63	RD	PHACO + PCIOL + 23-G-TSV + MP + EL + RT	SO-5000 cSt	LP	HM
19	F	73	TRD	PHACO + PCIOL + 23-G-TSV + MS + EL + RT	SO-5000 cSt	HM	CF
20	M	68	VH	PHACO + PCIOL + 23-G-TSV + EL	No	HM	20/200
21	M	74	VH	PHACO + PCIOL + 23-G-TSV	No	20/100	20/25
22	F	57	RD	PHACO + PCIOL + 23-G-TSV	SO-1000 cSt	CF	20/100
23	F	68	ERM	PHACO + PCIOL + 23-G-TSV + ERMP + İLMP	C_3_F_8_	20/200	20/60
24	M	18	RD	PHACO + PCIOL + 23-G-TSV	SO-1000 cSt	CF	20/100
25	M	61	ERM	PHACO + PCIOL + 23-G-TSV + MP	SO-1000 cSt	CF	20/100
26	F	55	RD	PHACO + PCIOL + 23-G-TSV + MP	SO-5000 cSt	HM	HM
27	F	57	TRD	PHACO + PCIOL + 23-G-TSV + MP	SO-1000 cSt	LP	HM
28	M	24	RD	PHACO + PCIOL + 23-G-TSV	SO-1000 cSt	CF	20/200

Preop.: Preoperative, postop.: postoperative, periop.: perioperative; BCVA: Best corrected visual acuity, F: female, M: male, VH: Vitreous hemorrhage, ERM: Epiretinal membrane, TRD: Tractional retinal detachment, RRD: Rhegmatogenous retinal detachment, IOFB: Intraocular foreign body, IOFBR: Intraocular foreign body removal, RS: retinoschisis, Phaco: phacoemulsification, PCIOL: Posterior chamber intraocular lens implantation, MP: Membrane peeling, CF: Counting fingers, HM: Hand motion, G: gauge, TSV: Transconjunctival sutureless vitrectomy, SO: Silicon oil, C_3_F_8_: perfluoropropane, SF_6_: Sulfur hexafluoride, RT: Retinal tear, IP: Iris prolapsus, ZD: Zonular dehiscence

Surgery was performed using general anesthesia or local anesthesia. A clear corneal incision was created at the temporal limbus. A 5- to 6-mm curvilinear capsulorhexis and coaxial phacoemulsification and irrigation/aspiration (Infinity Vision System, Alcon Inc., Fort Worth, TX, USA) were performed. A foldable intraocular lens was implanted into capsular bag. Corneal tunnel was closed with 10-0 nylon suture to prevent wound leakage and provide anterior chamber depth during vitrectomy. Preoperative indications, peri-operative and postoperative complications such as retinal detachment and posterior capsule opacification (PCO) formation, intraocular pressure changes, anatomical success, and visual results were recorded. After cataract surgery, the conjunctiva was displaced using a special pressure plate (DORC, Zuidland, Holland) over the intended sclerotomy sites. The cannula was then inserted at a 10°–30° angle through the conjunctiva, sclera, and pars plana 3.5 mm from the limbus. The cannulas were placed in the inferotemporal, superotemporal, and superonasal quadrants. The cannulas were inserted using a beveled trocaras a single-step procedure. An illumination probe was placed at the superonasal quadrant, and a 23-G infusion cannula was placed at the inferotemporal quadrant. A noncontact Biom indirect viewing system (Oculus Inc., Petaluma, CA) was used to visualization of the posterior segment.

Pars plana vitrectomy (PPV) was performed using a 23-G high-speed vitrector with a cut rate of 2500 per minute (Accurus Vitrectomy System, Alcon Inc., USA). The vitrectomy involved complete removal of the posterior vitreous till the vitreous base. The posterior hyaloid was removed using active aspiration in cases without complete posterior vitreous detachment. Triamcinolone acetonide was used to ensure that the posterior hyaloid was lifted and removed in all of the cases. The aspiration power was 300 mmHg, 500 mmHg, and 150 mmHg, in core vitrectomy, active aspiration of posterior hyaloid and removal of the peripheral posterior vitreous, respectively. After the fluid–air exchange, trypan blue was used in cases with epiretinal membrane (ERM) to facilitate membrane stripping and ERM was removed using 23-G microforceps. Endolaser treatment was applied with a curved 23-G laser probe (Iridex, Mountain View, CA). A posterior retinotomy with endodiathermy and an air-fluid exchange using a backflush brush were performed in the case of rhegmatogenous retinal detachment (RRD). Focal endolaser treatment was performed around the retinotomy site and retinal break. In cases with tractional retinal detachments (TRD), membrane dissection was carried out with the 23-G vitrectomy probe and hemostasis was yielded with 23-G endodiathermy. In cases with intraocular foreign body, the 20-G cannula was used for removal of foreign body following a partial vitrectomy performed with 23-G TSV. As endotamponade, perfluoropropane (*C_3_F_8_*) gas, sulfur hexafluoride (SF_6_) gas, air, or silicon oil were used. Silicone oil was injected with a 23-gauge cannula system and 10 mL injector with an injection pressure in complex cases such as RRD with inferiorly located breaks, proliferative vitreoretinopathy, or severe TRD. At the end of surgery, the cannulas were removed and the entry-site examined for leakage. Intraocular pressure was checked by palpation at the end of the operation.

Postoperative examinations were conducted at first day, first week and 1, 3, and 6 months, with a final visit at various times. Main outcomes were recorded including visual acuity, IOP, and both intraoperative and postoperative complications. Snellen visual acuity was converted into logarithm of the minimum angle of resolution (LogMar) for statistical analysis. Hypotony was defined as an IOP less than 6 mmHg. Preoperative indications, peri-operative and postoperative complications such as retinal detachment and posterior capsule opacification (PCO) formation, intraocular pressure changes, anatomical, and visual results were recorded.

All statistical analyses were performed using statistical software (SPSS for Windows, Version 16.0; SPSS, Inc., Chicago, IL) The Wilcoxon signed rank test was used to compare means with statistical significance threshold of *P* < 0.05.

## RESULTS

Twenty eight eyes of 28 patients were included in the case series. There were 15 right eyes (53.6%) and 13 left eyes (46.4%). Fifteen women (53.6%) and 13 men (46.4%), with a mean age of 59 ± 14 years (range, 18–74 years), were observed for a mean of 4.8 months, ranging from 3 to 15 months [Table 1].

The indications for surgery included ERM (8 eyes, 28.6%), RRD (7 eyes, 25%), diabetic-TRD (6 eyes, 21.5%), nonclearing vitreous hemorrhage (VH) (5 eyes, 17.9%) myopic macular retinoschisis (RS) (1 eye, 3.6%), and intraocular foreign body (IOFB) (1 eye, 3.6%) [Table 1]. All patients had clinically significant lens opacities. Surgery was performed using general anesthesia in 17 cases (60.7%) and local anesthesia in 11 cases (39.3%). A three-piece IOL was implanted in all eyes.

Intraoperative complications included non-entry-site iatrogenic retinal tears (3 eyes, 10.7%), retinal hemorrhage (1 eye, 3.6%), and prolapse of iris with zonular dehiscence (1 eye, 3.6%) [Table 2]. In the case with IOFB, a 20-G cannula was used for the removal of the foreign body following the partial 23-G TSV performed in the case that had an intraocular foreign body. This 20-G entry-site required suturing at the end of the surgery. No cases were converted to conventional 20-G vitrectomy. None of the cases required suture placement at the 23-G sclerotomy site. For intraocular tamponade, 13 patients had silicone oil (46.5%, 8 eyes 1000 centistokes (cSt), 5 eyes 5000 cSt), three had C_3_F_8_ (10.7%), two had SF6 (7.1%), four had air (14.3%), and six had no tamponade (21.4%) [Table 1].

**Table 2:** Intraoperative and one day postoperative complications

Complications	Rate
Preoperative	
Retinal tears	3/28
Retinal Hemorrhage	1/28
Iris prolapse and zonular dehiscence	1/28
Postoperative 1 day	
Hypotony (<6 mmHg)	3/28
Elevated IOP (> 21 mmHg)	4/28
Anterior chamber reaction	12/28
Corneal edema	10/28

IOP: intraocular pressure

**Figure 1:**
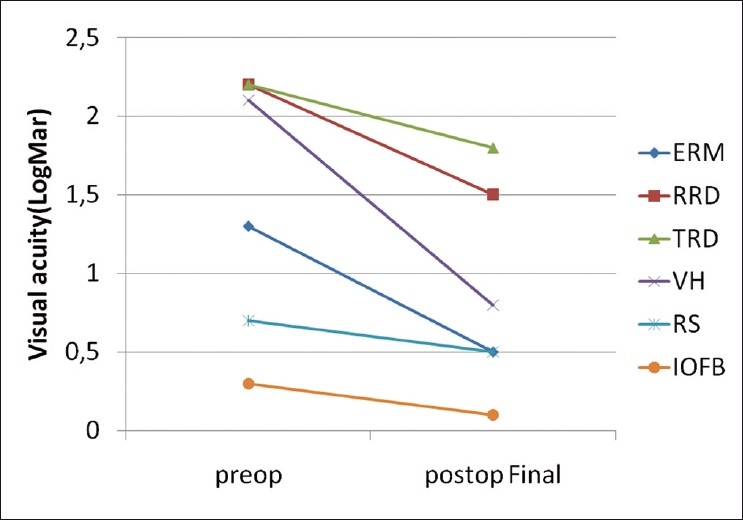
Visual acuity in logarithm of the minimal angle of resolution (LogMAR) units observed preoperatively and postoperatively for each vitreoretinal disease

Mean preoperative BCVA was 20/333 (range, 20/40 to light perception) and mean postoperative BCVA at final visit was 20/95 (range, 20/25 to light perception) [Figure 1], which was a statistically significant improvement (*P* < 0.01). Postoperative VA improved in 75% of patients (*P*<0,001) was reduced in 10.7%, and remained unchanged in 14.3

**Figure 2:**
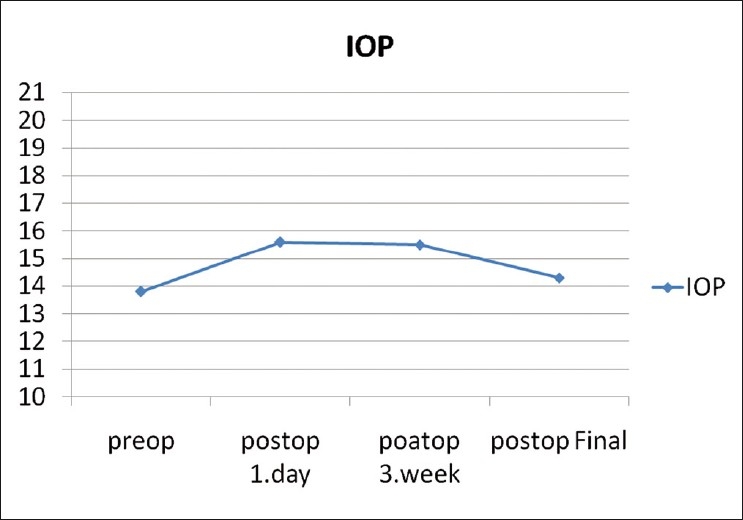
Preoperative and postoperative intraocular pressure changes at, 1 day, 3 week, and final postoperative visit

The mean preoperative IOP was 13.8 ± 3.3 mmHg, ranging from 6 to 19 mmHg. The mean postoperative IOP on day 1, week 1, and at the final visit was 15.6 ± 7.5 mmHg (range, 1-33 mmHg), 15.5 ± 6.7 mmHg (range, 8-36 mmHg), 14.3 ± 2.6 mmHg (range, 10-20 mmHg), respectively [Figure 2]. Compared with preoperative IOP, there was no significant difference in IOP on day 1, week 1, and at the final visit (*P* >0.05). One eye with SF6, 1 eye with silicone oil and 1 eye with air had hypotony at day one postoperatively [Table 2]. All of the three cases with hypotony improved by the first postoperative week. Four eyes with silicone oil (14.3%) (*P*=0,046) had IOP of more than 21 mmHg at day one postoperatively and required topical medication.

There was no complication related to general or local anesthesia. Postoperative complications at day one also included anterior chamber reaction ranging 2+ cells to fibrin formation (12 eyes, 42.8%) (*P*=0,001), and corneal edema (10 eyes, 35.7%) (*P*=0,002). Posterior capsular opacification developed in 3 eyes (11%) (*P*=0,08) [Table 2]. There was a correlation between postoperative anterior chamber reaction and PCO (*P*=0.03). During the follow-up period, no serious complications such as endophthalmitis or retinal detachment (RD) were noted.

## DISCUSSION

Combined vitreoretinal and cataract surgery has numerous advantages including less anesthesia risk, better intraoperative retinal visualization, performing sufficient peripheral vitrectomy, improving early visual rehabilitation, and the necessity of only one operation, which may reduce patient discomfort and decrease costs.^10^

However, simultaneous cataract and vitreoretinal surgery has potential disadvantages including difficulty in visualizing the capsulorrhexis due to an absent or reduced red reflex in cases with RD anda number of other potential complications.^8^,^10^,^11^

Combined 23 G-TSV and cataract surgery have been performed for numerous indications including vitreous hemorrhage, ERM, diabetic macular edema, macular hole, vitreomacular traction syndrome, diabetic TRD, and RRD.^9^,^12^

Others have reported that23-G TSV was reported safe and effective for cases with ERM. Hikichi *et al*. compared 20-G PPV and 23-G TSV in cases of ERM. The anatomical and functional outcomes were similar in both the groups.^13^ In our study, an ERM was the most common indication for surgery. (8 eyes, 28.6%). Anatomic success was obtained in 100% of eyes that underwent combined 23-G TSV and cataract surgery in cases with ERM. Postoperative BCVA improved in 75% of patients and remained unchanged in 25%.

Successful results were reported in cases with RRD repaired using 23 G-TSV. The RRD rate following 25-G TSV varies from 0-13%.^6^,^7^ In our series, 7 eyes with RRD underwent combined 23-G TSV and cataract surgery. Although the difficulties in intraocular manipulation had been experienced in our series, anatomic success was obtained in all of the cases. The removal of the peripheral vitreous up to the vitreous base could be meticulously applied the following cataract surgery. This is associated with the reduced rate of postoperative redetachment.

Fine *et al*. reported that of 12 patients who underwent 23 G-TSV for TRD, mean BCVA improved from 20/175 to 20/62.^7^ In our study, anatomic success was obtained in all cases that underwent surgery for TRD. In the 6 eyes with tractional retinal detachment, the mean preoperative BCVA was 20/2000 (range, light perception to count fingers) and the mean visual acuity at the last follow-up was 20/1000 (range, HM –20/200).

23-G TSV seems to be an effective procedure in the case of vitreous hemorrhage. A retrospective study of 10 eyes with vitreous hemorrhage demonstrated visual rehabilitation in 9 eyes.^14^ In our study, the causes of vitreous hemorrhage were branch retinal vein occlusion in two patients, diabetic retinopathy in two patients, and trauma in one patient. We found improved BCVA in all five vitreous hemorrhage cases.

We also used 23-G TSV and combined cataract surgery for two uncommon indications which incudedretinoschisis and IOFB. Successful results that had been reported in cases with retinoschisis following vitreoretinal surgery included core vitrectomy, surgically induced posterior vitreous detachment (PVD), with and without removal of the internal limiting membrane and gas tamponade.^15^,^16^ We did not find a case of retinoschisis performed using 23-G TSV. In our study, 23-G TSV including surgically induced PVD was performed in the case of retinoschisis. We obtained anatomic and functional success in cases using this procedure with the average visual acuity improving from 20/100 to 20/60.

We did not find a report of 23-G vitrectomy usage in a case with IOFB in the literature. In our case with IOFB, initially a partial 23-G vitrectomy was performed. Subsequently, a 23-G sclerotomy was converted to a 20-G sclerotomy for removal of IOFB.

The transient postoperative 1 day hypotony rate following 25-G TSV varies from 0% to 16.9%. The cause of hypotony may have been fluid leakage from the sclerotomy site.^12^ The better postoperative wound healing and less postoperative transient 1 day hypotony are obtained using angle incision in 23-G TSV. Postoperative 1 day hypotony was reported in 2 of 77 eyes by Fine *et al*. and in 1 of 24 eyes by Tsang *et al*.^4^,^7^ In our study, 3 eyes had transient postoperative hypotony. All of them improved by the first postoperative week. In 4 eyes with silicone oil, IOP was high. This was resolved with antiglaucoma therapy.

Chung *et al*. reported 13.5% of cases with anterior chamber fibrin exudation following 20-G vitrectomy and combined cataract surgery. However, they were not statistically significant. They reported that anterior chamber fibrin exudation occurred more frequently in the combined surgery group than in the sequential surgery group.^8^ In our study, we determined the anterior chamber inflammation in 42.8% of eyes, and corneal edema in 35.7%.

PCO may develop after cataract surgery and our rate was 11% which was comparable to that reported in a study of Sood *et al*^9^ requiring Neodymium:YAG capsulotomy developed in 8 of 60 eyes (13.3%).^9^ which was similar to tha reported by Sood *et al*. All of these cases with PCO underwent gas tamponade. The PCO formation may be associated with the intraocular inflammation due to these cases had anterior chamber reaction in the early postoperative period. Neodymium:YAG capsulotomy was performed in these cases.

Another important complication in TSV is endophthalmitis. A study reported that the rate of endophthalmitis following 20 G-PPV was 0.018%, and 0.23% following 25-G TSV.^17^ The high rate with 25-G TSV was associated with contributing factors including a lack of wound closure, postoperative hypotony, and lower fluid infusion rates. However, the actual risk for endophthalmitis in 23 G-TSV with and without combined cataract surgery is unknown.^9^ There were no cases of endophthalmitis in our study.

23-G TSV has a number of potential disadvantages. Smaller port and smaller diameter vitreous cutters may decrease cutting and aspiration rates. With the vitreous cutting system we used, we achieved flow rates compared to a conventional 20-gauge system.^9^ Currently, there is no fragmatome smaller than 20-G. Although cortical and small nuclear lenticular fragments may be removed with a 23-G cutter, large nuclear pieces and certain intraocular foreign bodies require at least one 20-G sclerotomy. In our study, a 20-G sclerotomy was required for removal of the foreign body following partial 23 G-TSV. The cannulas are more difficult to insert than in 25-G systems because of the additional steps required. There are fewer 23-G instruments commercially available than 25-G.^7^

In conclusion, combined cataract surgery and transconjunctival sutureless vitrectomy is a safe and effective option in patients with significant lens opacities and vitreoretinal diseases. Combined cataract and vitreous surgery is more convenient, and is likely more cost-effective compared to sequential cataract and vitrectomy surgery.
